# Cefazolin prophylaxis is associated with lower surgical site infection risk than vancomycin following elective spine surgery

**DOI:** 10.1016/j.xnsj.2026.100857

**Published:** 2026-02-08

**Authors:** Michael S. Kim, Melissa R. Romoff, Ryan Beyer, Emily Tse, Hao-Hua Wu, Don Y. Park, Yu-Po Lee, Nitin Bhatia, Sohaib Hashmi, Emily S. Mills

**Affiliations:** Department of Orthopedic Surgery, University of California, Irvine, School of Medicine, 101 The City Dr S, Pavilion 3, Building 29A, Orange, CA 92868, United States

**Keywords:** Cefazolin, Vancomycin, Surgical site infection, Spine surgery, Antibiotic prophylaxis, Anti-bacterial agents

## Abstract

**Background:**

Surgical site infection (SSI) remains a major cause of morbidity following elective spine surgery, with incidence rates up to 3%. Current guidelines recommend cefazolin as the first-line prophylactic antibiotic due to its efficacy against methicillin-sensitive *Staphylococcus aureus* (MSSA) and coagulase-negative staphylococci (CoNS). However, reported B-lactam allergies are commonly cited in clinical practice as a reason for substitution with vancomycin, which provides inferior coverage for MSSA and limited gram-negative activity. Prior single center studies suggest higher SSI risk with vancomycin, but large-scale evidence remains limited.

**Methods:**

A retrospective cohort analysis was conducted using the TriNetX US Collaborative Network (2015-2023). Adults undergoing elective spine surgery, including transforaminal or posterior interbody fusion (TLIF/PLIF), multilevel lumbar fusion, posterior cervical fusion, anterior cervical discectomy and fusions (ACDF), and microdiscectomy, were identified. Patients receiving perioperative cefazolin or vancomycin within 24 hours of surgery were included. 1:1 propensity score matching controlled for demographics, comorbidities, and infection risk factors. The primary outcome was 90-day SSI, assessed using ICD-10 diagnosis codes. Risk ratios (RR) were estimated using Cox proportional hazards models.

**Results:**

After matching, 12,996 patients were included across 5 procedure cohorts. Cefazolin prophylaxis was associated with lower or comparable SSI risk compared with vancomycin across all procedures. Statistically significant differences were observed for TLIF/PLIF (0.82% vs. 2.30%; RR 0.36, 95% CI 0.18–0.7; p = .0019) and microdiscectomy (0.34% vs. 0.69%; RR 0.50, 95% CI 0.27–0.93; p = .025). Nonsignificant trends favored cefazolin for posterior cervical fusion (0.88% vs. 1.41%), multilevel lumbar fusion (1.25% vs. 1.51%), and ACDF (0.30% vs. 0.54%).

**Conclusions:**

Cefazolin prophylaxis is associated with significantly lower SSI risk in elective spine procedures. These findings support cefazolin as the preferred perioperative antibiotic and emphasize the need for accurate B-lactam allergy assessment to optimize infection prevention and antimicrobial stewardship in spine surgery.

## Introduction

Postoperative surgical site infection (SSI) remains a major source of morbidity a cost following elective spine surgery, with incidence rates ranging from 0.5% to 3% and annual U.S. expenditures exceeding $3 billion [1,2]. Prophylactic antibiotics are a cornerstone of SSI prevention, and current Infectious Diseases Society of American (IDSA) and North American Spine Society (NASS) guidelines recommend first-generation cephalosporins, particularly cefazolin, as the preferred perioperative agent [[3], [4], [5]]. Cefazolin offers a potent bactericidal activity against methicillin-sensitive *Staphylococcus aureus* (MSSA) and coagulase-negative *Staphylococci* (CoNS), which predominate in spine SSIs [[6], [7], [8]], while providing favorable tissue penetration and safety [9]. Vancomycin and clindamycin are reserved for patients with confirmed B-lactam allergy or MRSA colonization [3,5,10].

However, the accuracy of B-lactam allergy labeling represents a major challenge in antimicrobial stewardship. Although 8% to 20% of patients report such an allergy, over 90% can safely receive cefazolin upon formal evaluation [10,11]. Substitution with broader-spectrum alternatives such as vancomycin has been consistently associated with higher SSI rates across multiple orthopedic subspecialties. In arthroplasty, Wyles et al. [12] demonstrated that non-cefazolin regimens doubled prosthetic joint infection risk, while implementing preoperative allergy testing improved cefazolin use and reduced infection. Similar findings have been reported in hip and knee arthroplasty and trauma cohorts, where cefazolin prophylaxis provided superior protection against *Staphylococcus* species compared with vancomycin or clindamycin [[13], [14], [15]].

Within spine surgery, several institutional and regional studies have reported higher SSI rates when cefazolin is replaced by alternative agents, particularly vancomycin [[16], [17], [18]]. In a large single-center retrospective cohort of over 10,000 primary spine procedures, patients receiving non-cefazolin antibiotics, most commonly vancomycin due to reported penicillin allergy, experienced more than a twofold increase in postoperative infection risk compared with those receiving cefazolin, even after adjustment for BMI and operative factors [16]. Similarly, analyses of elective multilevel posterior spinal fusion have demonstrated vancomycin prophylaxis to be independently associated with increased odds of both superficial and deep SSI, alongside known risk factors such as longer operative time and revision surgery [18]. Beyond single institution studies, a multicenter registry study from Japan leveraged a natural experiment where a national cefazolin shortage was created and found that substitution with alternative broad-spectrum agents was associated with nearly double the risk of reoperation for deep SSI, particularly in thoracolumbar procedures [17]. Despite consistent signals across these studies, their reliance on single-center or limited regional cohorts, procedure-specific populations, and relatively small numbers of infectious events limits generalizability and precludes definitive conclusions regarding national practice patterns and outcomes associated with perioperative antibiotic selection in spine surgery.

To date, no large-scale national study has evaluated the association between perioperative antibiotic choice and postoperative SSI risk across a broad spectrum of elective spine procedures. Accordingly, we leveraged a large national database to examine perioperative antibiotic selection and 90-day SSI outcomes following elective spine surgery. Our goal was to determine whether previously reported institutional trends favoring cefazolin are upheld at the national level. We hypothesized that cefazolin prophylaxis would be associated with significantly lower SSI risk compared with non-cefazolin regimens, consistent with findings across other orthopedic subspecialties.

## Methods

### Study design and database

This study was a retrospective cohort analysis utilizing the TriNetX US Collaborative Network, a federated database that aggregates de-identified electronic health records from healthcare organizations across the United States, representing over 117 million patients. TriNetX complies with HIPAA standards for data security and confidentiality, and all data are fully de-identified; therefore, this study was exempt from IRB approval.

### Cohort selection

Patients aged 18 years or older who underwent elective spine surgery between 2015 and 2023 were identified using Current Procedural Terminology (CPT) procedure codes for posterior lumbar interbody fusion (PLIF), transforaminal lumbar interbody fusion (TLIF), microdiscectomy, posterior cervical fusion, anterior cervical discectomy and fusion (ACDF), and multilevel lumbar fusion (3–6 levels) (Supplementary Material Table 1). The operative date served as the index event.

Patients were included if they received prophylactic postoperative antibiotics (cefazolin, vancomycin, or clindamycin) administered on the day of surgery or within the first 7 postoperative days. Because incision-level antibiotic timing is not reliably captured in TriNetX, this window was selected to identify perioperative and early postoperative prophylactic antibiotic selection patterns documented in routine clinical practice.

Patients were excluded if they were younger than 18 years; underwent surgery for trauma, tumor, or infection-related indications; received an antibiotic other than cefazolin, vancomycin, or clindamycin, received more than one of the study antibiotics; had systemic antibiotic exposure in the 30 days prior to surgery; or received any of the study antibiotics beyond postoperative day 7 (postoperative days 8–30). These criteria were applied to minimize misclassification of therapeutic antibiotic use and to isolate perioperative and early postoperative prophylactic antibiotic selection.

Although clindamycin was included as an eligible prophylactic agent to reflect guideline-recommended alternatives for patients with reported B-lactam allergy, the number of patients receiving clindamycin who met all inclusion and exclusion criteria was insufficient to support robust propensity score matching or procedure-specific comparative analysis. Therefore, propensity score matching and primary analyses were limited to cefazolin and vancomycin cohorts. Clindamycin recipients were excluded from matched comparisons.

### Propensity score matching

To reduce confounding, 1:1 propensity score matching (PSM) was performed between antibiotic cohorts using TriNetX’s built-in-nearest-neighbor algorithm. Matching covariates included demographic factors (age, sex, body mass index), comorbidities (diabetes mellitus, chronic kidney disease, peripheral vascular disease), and clinical risk factors (smoking status, chronic corticosteroid use, immunosuppressant use, prior methicillin-resistant *S. aureus* [MRSA] infection). Standardized mean differences (SMDs) were calculated, with <0.1 indicating acceptable balance between groups. Postmatching cohort characteristics are reported in Table 1, Table 2, Table 3, Table 4, Table 5.

**Table 1:** Postmatching characteristics ACDF (cefazolin vs. vancomycin).

*Characteristic*	*Cefazolin (*N *=4,051)*	*Vancomycin (*N *=4,051)*	p*-value*	*SMD*
Age (mean ± SD)	62.5 ± 5.7	62.5 ± 5.4	.8701	0.0000
Sex				
Male	1,522 (38.29%)	1,533 (38.97%)	.8009	0.0110
Female	2,206 (54.46%)	2,216 (54.70%)	.8234	0.0051
Labs				
BMI (mean ± SD)	32.6 ± 7.34	28.9 ± 5.16	.1303	0.0150
MRSA	324 (7.89%)	340 (8.39%)	.5170	0.0024
Diagnosis				
Diabetes mellitus	809 (19.97%)	818 (20.13%)	.8029	0.0083
Nicotine dependence	838 (20.69%)	815 (20.12%)	.5260	0.0241
Chronic kidney disease	239 (5.90%)	268 (6.16%)	.1834	0.0240
Long term (current) uses of steroids	138 (3.41%)	175 (4.32%)	.0329	0.0471
Other peripheral vascular diseases	187 (4.62%)	201 (4.96%)	.4664	0.0164
Medications				
Immunosuppressants	258 (6.37%)	264 (6.52%)	.7860	0.0211

**Table 2:** Postmatching characteristics lumbar 3–6 (cefazolin vs. vancomycin).

*Characteristic*	*Cefazolin (*N *=4,051)*	*Vancomycin (*N *=4,051)*	p*-value*	*SMD*
Age (mean ± SD)	71.5 ± 3.4	71.3 ± 2.8	.5066	0.0181
Sex				
Male	809 (53.12%)	813 (53.38%)	.8845	0.0050
Female	606 (39.79%)	606 (39.79%)	1.0000	0.0000
Labs				
BMI (mean ± SD)	34.6 ± 11.5	29.4 ± 5.26	.2069	0.0542
MRSA	40 (2.63%)	41 (2.69%)	.0239	0.0043
Diagnosis				
Diabetes mellitus	412 (27.05%)	403 (26.46%)	.7126	0.0134
Nicotine dependence	244 (16.02%)	250 (16.42%)	.7681	0.0111
Chronic kidney disease	122 (8.01%)	140 (9.192%)	.2448	0.0412
Long term (current) uses of steroids	100 (6.57%)	111 (7.29%)	.4325	0.0288
Other peripheral vascular diseases	89 (5.84%)	109 (7.16%)	.1416	0.0287
Medications				
Immunosuppressants	95 (6.24%)	113 (7.42%)	.1960	0.0541

**Table 3:** Postmatching characteristics posterior cervical (cefazolin vs. vancomycin).

*Characteristic*	*Cefazolin (*N *=4,051)*	*Vancomycin (*N *=4,051)*	p*-value*	*SMD*
Age (mean ± SD)	68.1 ± 4.5	67.9 ± 5.3	.5782	0.0202
Sex				
Male	831 (48.71%)	824 (48.30%)	.8105	0.0088
Female	814 (47.71%)	815 (47.77%)	.9727	0.0014
Labs				
BMI (mean ± SD)	29 ± 6.87	31.7 ± 4.37	.1054	0.0461
MRSA	74 (4.34%)	75 (4.34%)	.9803	0.000
Diagnosis				
Diabetes mellitus	475 (27.84%)	475 (27.84%)	1.0000	0.0071
Nicotine dependence	325 (19.05%)	330 (19.34%)	.8279	0.0064
Chronic kidney disease	191 (11.20%)	188 (11.02%)	.8702	0.0122
Long term (current) uses of steroids	70 (4.10%)	74 (4.34%)	.7334	0.0280
Other peripheral vascular diseases	106 (6.21%)	118 (6.92%)	.4068	0.0101
Medications				
Immunosuppressants	143 (8.38%)	138 (8.10%)	.7555	0.0133

**Table 4:** Postmatching characteristics microdiscectomy (cefazolin vs. vancomycin).

*Characteristic*	*Cefazolin (*N *=4,051)*	*Vancomycin (*N *=4,051)*	p*-value*	*SMD*
Age (mean ± SD)	58.5 ± 6.4	58.5 ± 5.8	.9697	0.000
Sex				
Male	1,956 (44.76%)	1,951 (44.65%)	.9143	0.0021
Female	2,139 (48.95%)	2,142 (49.02%)	.9488	0.0017
Labs				
BMI (mean ± SD)	31.6 ± 4.47	31.9 ± 7.76	.1108	0.0466
MRSA	89 (2.04%)	109 (2.49%)	.0029	0.0301
Diagnosis				
Diabetes mellitus	608 (13.91%)	604 (13.82%)	.9015	0.0032
Nicotine dependence	573 (13.11%)	579 (13.25%)	.8279	0.0043
Chronic kidney disease	184 (4.21%)	187 (4.28%)	.8735	0.0031
Long term (current) uses of steroids	112 (2.56%)	127 (2.91%)	.3252	0.0218
Other peripheral vascular diseases	131 (2.99%)	125 (2.86%)	.7035	0.0081
Medications				
Immunosuppressants	207 (4.74%)	201 (4.60%)	.7610	0.0078

**Table 5:** Postmatching characteristics TLIF/PLIF (Cefazolin vs. Vancomycin).

*Characteristic*	*Cefazolin (*N *=4,051)*	*Vancomycin (*N *=4,051)*	p*-value*	*SMD*
Age (mean ± SD)	68.5 ± 3.5	68.2 ± 5.1	.4563	0.2110
Sex				
Male	538 (39.97%)	546 (40.57%)	.7532	0.0125
Female	728 (54.09%)	718 (53.34%)	.6991	0.0157
Labs				
BMI (mean ± SD)	28.3 ± 8.27	32.4 ± 8.83	.3507	0.0477
MRSA	62 (4.61%)	74 (5.50%)	.1130	0.0408
Diagnosis				
Diabetes mellitus	338 (25.11%)	333 (24.74%)	.8237	0.0096
Nicotine dependence	216 (16.05%)	221 (16.42%)	.7938	0.0104
Chronic kidney disease	113 (8.34%)	125 (9.29%)	.8735	0.0337
Long term (current) uses of steroids	73 (5.42%)	78 (5.80%)	.6754	0.0164
Other peripheral vascular diseases	87 (6.46%)	98 (7.28%)	.4020	0.0322
Medications				
Immunosuppressants	71 (5.28%)	87 (6.46%)	.1895	0.0503

Because several intraoperative and process-of-care variables relevant to SSI risk (ex: operative duration, blood loss, redosing, wound class, and topical vancomycin powder use) are not consistently available in TriNetX, confounding was mitigated using procedure-specific cohorting and propensity score matching on major patient-level SSI risk factors reliably captured across sites.

### Statistical analysis

The primary outcome was postoperative infection, defined by an ICD diagnosis code for surgical site infection or deep wound infection within 90 days of the index procedure (Supplementary Material Table 1). Absolute risks were calculated as event counts and percentages. Risk ratios (RR) were estimated using Cox proportional hazards models. All analyses were performed within the TriNetX integrated analytic platform, with a significance threshold of p < .05.

## Results

Following propensity score matching, a total of 12,996 patients were included across 5 elective spine procedure cohorts: 1,346 undergoing TLIF/PLIF, 4,370 microdiscectomy, 1,706 posterior cervical fusion, 1,523 multilevel lumbar fusion (3–6), and 4,051 ACDF (Table 1, Table 2, Table 3, Table 4, Table 5).

**Fig. 1:**
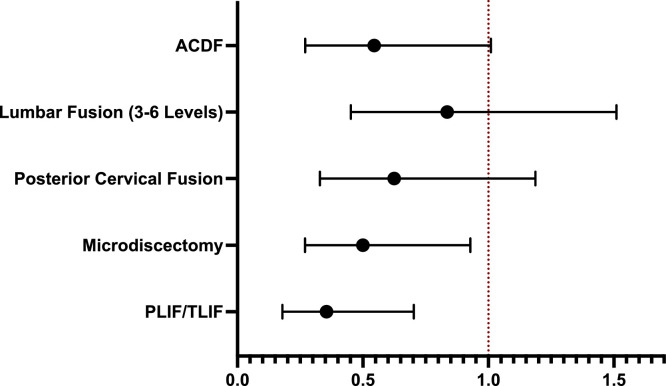
Relative risk of surgical infection with cefazolin versus vancomycin across spine procedures. Forest plot showing relative risk and 95% confidence intervals for surgical site infection comparing cefazolin to vancomycin across 5 spine procedure types. Values less 1.0 indicate reduced infection risk with cefazolin. The vertical dashed line represents RR = 1.0 (no difference).

Across all procedures, cefazolin demonstrated lower or comparable postoperative infection rates relative to vancomycin (Table 6, Figure 1). Statistically significant differences were observed in the PLIF/TLIF and microdiscectomy cohorts. In PLIF/TLIF, infection occurred in 0.82% of patients receiving cefazolin compared with 2.30% of patients receiving vancomycin, corresponding to a 65% relative risk reduction (RR 0.36; 95% CI 0.18–0.70; p = .0019). Similarly, among microdiscectomy patients, cefazolin was associated with significantly fewer infections (0.34% vs. 0.69%; RR 0.50, 95% CI 0.27–0.93, p = .025).

**Table 6:** Comparison of cefazolin versus vancomycin for surgical site infection (SSI) across spine procedures.

*Procedure type*	n	*SSI rate (Cefazolin)*	*SSI rate (Vancomycin)*	*Relative risk (95% ci)*	p*-value*
PLIF/TLIF	1,346	0.82%	2.30%	0.355 (0.179–0.703)	.0019
Microdiscectomy	4,370	0.34%	0.69%	0.500 (0.269–0.928)	.0250
Posterior cervical fusion	1,706	0.88%	1.41%	0.625 (0.329–1.187)	.1470
Lumbar fusion (3–6 levels)	1,523	1.25%	1.51%	0.826 (0.452–1.51)	.5340
ACDF	4,051	0.30%	0.54%	0.545 (0.27–1.01)	.0860

In posterior cervical fusion, cefazolin recipients experienced lower infection rates compared to vancomycin (0.88% vs. 1.41%), though this difference did not reach statistical significance (RR 0.63, 95% CI 0.33–1.10; p = .147). Likewise, no significant differences were observed in multilevel lumbar fusion (1.25% vs. 1.51%; RR 0.83, 95% CI 0.45–1.50, p = .534) or ACDF (0.30% vs. 0.54%; RR 0.55, 95% CI 0.27–1.01, p = .086), although trends consistently favored cefazolin.

Comparisons with clindamycin were not performed due to insufficient patient numbers after applying inclusion and exclusion criteria.

## Discussion

In our analysis of 12,996 elective spine procedures, cefazolin prophylaxis was associated with lower or comparable SSI risk compared with vancomycin across all procedure types. The association reached statistical significance for PLIF/TLIF and microdiscectomy, while posterior cervical fusion, multilevel lumbar fusion, and ACDF showed similar but nonsignificant trends. These findings validate prior single-center results using a large, multicenter U.S. database encompassing hundreds of hospitals and surgeons, strengthening the evidence that cefazolin provides optimal prophylactic coverage in spine surgery and emphasizing the importance of appropriate allergy evaluation and antibiotic selection.

The microbiology of spine SSIs further supports these findings. MSSA and CoNS remain the predominant pathogens, for which cefazolin provides potent bactericidal activity [3]. Vancomycin, while valuable for MRSA coverage, is less effective against MSSA and lacks gram-negative activity, which may contribute to the higher SSI risk observed with its use.

The magnitude and statistical significance of observed differences across procedure types reflect both underlying procedural risk and available statistical power. Higher-risk, higher-volume procedures such as TLIF/PLIF are characterized by longer operative times, greater blood loss, and routine instrumentation, resulting in higher baseline SSI rates and greater number of infectious events, thereby increasing power to detect differences between antibiotic groups [[19], [20], [21], [22], [23]]. In contrast, procedures with lower baseline SSI rates, such as microdiscectomy and ACDF, yielded fewer infection events despite large cohort sizes, limiting statistical power even when effect estimates consistently favored cefazolin [3,[24], [25], [26], [27]]. Thus, variability in statistical significance across procedures likely reflects differences in event rates and power rather than biologically divergent effects of antibiotic selection.

In the present study, cefazolin-based perioperative antibiotic selection was associated with a 50% to 65% relative reduction in 90-day SSI risk compared to vancomycin in higher-risk procedures, with effect estimates consistently favoring cefazolin across all evaluated spine surgery categories.

Several recent studies have evaluated infection prophylaxis choice in spine surgery, though most were limited by single-center design. Kazarian et al. [13] conducted a retrospective analysis of 10,122 spine surgeries at a single U.S. academic institution, finding that vancomycin prophylaxis increased infection risk 2.5-fold compared with cefazolin (OR 2.48, p = .017). Their cohort encompassed all spine procedures collectively, without procedure-specific stratification. Similarly, Nakarai et al. [17] examined 3,841 spine operations across 4 Japanese centers during a national cefazolin shortage, observing nearly double the risk of reoperation for infection when alternative B-lactam agents were used (OR 1.96, p = .014). Herrington et al. analyzed 535 patients undergoing posterior thoracolumbar multilevel fusions and found that vancomycin prophylaxis was associated with a 2.5-fold increased infection risk (OR 2.50; p = .031) [18]. However, their study population was restricted to a single center and a narrow surgical subset. Our study expands upon these findings using a multicenter national U.S. database comprising hundreds of hospitals and surgeons, and by performing separate analysis for distinct procedure types, thereby providing my granular insight into infection risk patterns across surgical categories.

This study has several limitations inherent to retrospective analyses. First, while we performed a propensity score-matched analysis controlling for patient demographics as well as many risk factors for infection, important intraoperative and process-of-care factors known to influence SSI, including operative duration estimated blood loss, wound class, antibiotic infusion timing and redosing, intrawound vancomycin powder use, wound drains, and surgeon or hospital volume, are not consistently available across institutions in TriNetX network and therefore and could not be included in matching. In addition, SSI outcomes were defined using ICD diagnostic codes, which may be subject to misclassifications or coding inaccuracies inherent to administrative data sources.

To mitigate confounding related procedural complexity and baseline infection risk, we performed analyses within procedure-specific cohorts, which partially proxy for differences in invasiveness, instrumentation, and operative duration, and applied propensity score matching on major patient-level SSI risk factors reliably across sites. Nevertheless, residual unmeasured confounding my persist.

Additionally, although prior MRSA infection was included as a matching variable, this does not fully capture MRSA colonization status at the time of surgery. Preoperative MRSA screening results (e.g., nasal colonization) are not uniformly available within TriNetX and therefore could not be directly assessed. As a result, residual confounding related to unmeasured MRSA colonization risk may remain.

Importantly, in routine clinical practice, selection of vancomycin over cefazolin is frequently driven by reported B-lactam allergy or perceived MRSA risk, factors that are themselves associated with increased baseline infection risk and greater medical complexity. As a result, patients receiving vancomycin often represent a higher-risk population at baseline. Consequently, any unmeasured confounding would be expected to bias estimates toward higher observed SSI risk among vancomycin recipients rather than generate a protective association for cefazolin.

In summary, our results support cefazolin as the first-line postoperative prophylactic antibiotic in elective spine surgery. Vancomycin should be reserved for patients with confirmed MRSA colonization or verified severe B-lactam allergy, ideally after formal preoperative allergy evaluation to allow safe delabeling when possible. Future efforts should focus on implementing cefazolin-first protocols and studying systematic allergy delabeling workflows to optimize antimicrobial stewardship and SSI prevention in spine surgery.

## Conclusion

Across 12,996 matched procedures, cefazolin demonstrated lower or equivalent infection rates compared with vancomycin, with statistically significant reduction in PLIF/TLIF and microdiscectomy. These findings reinforce national guideline recommendations and highlight the risks associated with substitution of cefazolin with alternative agents. Broader implementation of cefazolin-first protocols, supported by accurate allergy assessment strategies, could meaningfully reduce postoperative infections and advance microbiology stewardship in spine surgery.

## Declaration of competing interest

The authors declare that they have no known competing financial interests or personal relationships that could have appeared to influence the work reported in this paper

## Appendix Supplementary materials

Image, application 1
