# Supplementary material for: Cefazolin prophylaxis is associated with lower surgical site infection risk than vancomycin following elective spine surgery
Source: N Am Spine Soc J. 2026 Feb 8;25:100857. doi: 10.1016/j.xnsj.2026.100857 (PMC12995863; doi:10.1016/j.xnsj.2026.100857)
Supplement: Supplementary file 1 [file mmc1.docx]

Supplementary Figure 1 CPT Codes

ACDF:

22551, 22554

Lumbar Fusion (3-6 Levels):

22612, 22842

Posterior Cervical Fusion:

22600

Microdiscectomy:

63030

PLIF/TLIF:

22633, 22630, 22842
